# Effects of Extreme Temperature Stress on the Gut Microbiota of Light-Vented Bulbul (*Pycnonotus sinensis*)

**DOI:** 10.3390/biology15212045

**Published:** 2026-11-09

**Authors:** Ziyi Wang, Qi Yang, Guangfei Guo, Yizhou Zhang, Mingwen Duan, Youyang Chen, Xiaoying Xing, Ying Xiong, Hongtao Xiao

**Affiliations:** 1 Department of Zoology, College of Life Sciences, Sichuan Agricultural University, Ya’an 625000, China; 2 Key Laboratory of Animal Physiology, Biochemistry and Molecular Biology of Hebei Province, College of Life Sciences, Hebei Normal University, Shijiazhuang 050024, China; 3 College of Wildlife and Protected Area, Northeast Forestry University, Harbin 150040, China

**Keywords:** light-vented bulbul, temperature treatment, gut microbiota, bacterial diversity 16S rRNA gene sequencing

## Abstract

Temperature variation is a key environmental factor affecting the physiology and ecology of wild birds; however, experimental evidence for how sustained temperature exposure shapes the gut microbiota of wild passerines remains limited. In this study, 18 adult light-vented bulbuls (*Pycnonotus sinensis*) were randomly assigned to low temperature (LT, 0 °C), room temperature (RT, 24 °C), or high temperature (HT, 40 °C) groups for 60 days. Fecal microbiota were profiled by 16S rRNA gene amplicon sequencing of the V3–V4 region. After quality filtering and removal of non-bacterial sequences, 1490 amplicon sequence variants (ASVs) were retained. Firmicutes and Proteobacteria dominated all samples, together accounting for more than 90% of the relative abundance, with no significant differences among groups. At the genus level, Candidatus *Arthromitus* was most abundant in all groups and peaked in the LT group (75.39%), whereas *Ligilactobacillus* was significantly enriched in the HT group relative to the RT group (19.68%, adjusted *p* = 0.028). Alpha diversity analyses showed that observed ASVs and Faith’s phylogenetic diversity were significantly higher in the LT group than in the RT and HT groups, whereas Shannon, Simpson, and Pielou evenness did not differ significantly among groups. Beta diversity based on Aitchison distances revealed a significant shift in community structure among treatments (PERMANOVA, *R*^2^ = 0.326, *p* = 0.014), with no significant difference in within-group dispersion (PERMDISP, *p* = 0.559). LEfSe identified 25 differentially abundant genera, 16 of which were enriched in the LT group, while PICRUSt2-based functional prediction detected no significant differences in KEGG pathways after FDR correction. These results indicate that sustained temperature exposure, particularly low temperature, reshapes the gut microbiota of light-vented bulbuls primarily at the genus level and increases richness and phylogenetic diversity, whereas predicted functional profiles remain similar among treatments.

## Simple Summary

This study examined how 60 days of exposure to low, room, and high temperatures affected the gut microbiota of a wild passerine, the light-vented bulbul (*Pycnonotus sinensis*). Using controlled low-, room-, and high-temperature treatments combined with 16S rRNA gene sequencing, we showed that temperature reshapes the gut bacterial community primarily at the genus level. Low temperatures increased both observed amplicon sequence variant (ASV) richness and phylogenetic diversity, while high temperatures were associated with a marked enrichment of *Ligilactobacillus*. The dominant phyla, Firmicutes and Proteobacteria, remained stable across treatments, and no significant differences in predicted KEGG pathways were detected. These findings provide controlled experimental evidence that temperature, particularly cold exposure, can restructure the gut microbiota of wild birds, and highlight the value of combining taxonomic and functional analyses in future studies of host–microbe interactions under climate change.

## 1. Introduction

Global climate change has made temperature variation a critical environmental factor affecting the physiology and ecology of wild animals [1,2]. Temperature variation not only influences metabolic rates and energy budgets [3,4], but also indirectly shapes ecological processes and reproductive activities by altering habitat conditions, food resources, and offspring provisioning [5,6]. Birds respond to environmental change at physiological, behavioral, and population levels, and some species are used as bioindicators for ecosystem monitoring [7]. In response to temperature variation, birds make behavioral and physiological adjustments, including range shifts [8,9,10], changes in metabolic capacity and thermoregulation [3,4,11,12,13,14,15], and shifts in breeding phenology and parental provisioning behavior [5,6,16]. These responses involve both metabolic heat production and evaporative heat loss and are modulated by food availability and habitat conditions [11,12,15]. For example, climate warming is associated with advanced breeding phenology in some birds [6,16] and poleward or elevational range shifts [8,9,10]. High temperatures can also alter parental food-supply behavior and energy allocation during reproduction [5].

The gut microbiota participate in host responses to temperature change and offer new insights into environment–host physiological interactions [17,18,19]. Often called the host’s “second genome” [20,21], the gut microbiome plays important roles in nutrient utilization, metabolism, and immune regulation, which are central themes in avian microbiome research [22,23,24]. Temperature can reshape gut communities by modifying microbial growth conditions and host physiological states, and these effects vary among host species [17,18,25]. Studies of heat-stressed poultry also show that high temperatures alter cecal and ileal microbial composition and are linked to host enteric serotonin responses, the regulation of thermogenesis, and changes in feed intake [26,27,28,29,30]. A manipulative field experiment on wild birds found that nest warming and parasitism had divergent effects on the gut microbiota of Eastern bluebirds and tree swallows [31]. Even under a constant diet, homing pigeons show summer–winter gut microbial divergence, suggesting that temperature and other seasonal drivers contribute to community turnover [25]. Large-scale field surveys of passerines have documented seasonal shifts in gut microbial diversity and composition [32]. Studies of tree sparrows suggest that gut microbiota are relevant to host adaptation to high-altitude environments [33]. Together, these findings indicate that avian gut microbial responses to temperature should be interpreted in light of host taxonomy, seasonal context, and exposure conditions [17,18,25,31,32,33].

Although comparative field data on avian gut microbiomes are accumulating, seasonal and geographical surveys cannot disentangle temperature from confounding factors such as food availability and photoperiod [25,32]. Therefore, comparisons under standardized captive conditions with long-term high- and low-temperature treatments can clarify links between temperature and gut community profiles and help assess whether taxonomic shifts are accompanied by detectable changes in predicted function [17,25,34]. The light-vented bulbul (*Pycnonotus sinensis*) has markedly expanded its range northward in recent decades, making it a suitable model for studying avian physiological and microbial responses to different thermal environments [10,35]. Previous studies have shown seasonal phenotypic plasticity in body mass, organ size, and tissue oxidative capacity in this species [36]; temperature and photoperiod treatments can also alter its energy intake and thermoregulation [14,15]. In addition, the gut microbiota of northward-expanding populations are correlated with host resting metabolic rate [35]. However, this physiological and correlative field evidence is insufficient to determine gut microbial responses under sustained temperature manipulation. We therefore conducted low-, room-, and high-temperature treatments combined with 16S rRNA gene sequencing and functional prediction. We analyzed fecal bacterial community composition, diversity, and predicted functional profiles of *P. sinensis* to provide community-level experimental evidence for temperature-mediated host–microbe interactions [17,24,34].

## 2. Materials and Methods

### 2.1. Experimental Animals and Housing

Eighteen healthy adult light-vented bulbuls (*Pycnonotus sinensis*) were captured on the campus of Sichuan Agricultural University in October 2024. All birds were acclimated under laboratory conditions at 24 °C for 7 days. Afterwards, individuals were stratified by initial body mass and randomly allocated within strata to three groups with six birds per group. Baseline fecal samples (day 0) prior to the experimental treatment were not collected. No significant inter-group difference in initial body mass was observed (low-temperature group: 29.06 ± 3.33 g; room-temperature control group: 29.03 ± 2.08 g; high-temperature group: 28.74 ± 1.30 g; one-way ANOVA: F(2,15) = 0.033, *p* = 0.968). Birds were maintained under three temperature treatments—low temperature (LT, 0 °C), room temperature (RT, 24 °C), and high temperature (HT, 40 °C)—for 60 days under a standardized 12 h light–dark photoperiod (08:00–20:00). Each treatment group was housed in a separate chamber containing three cages (two birds per cage); relative humidity was maintained at 50–60%, and temperature was held at the target value. Fresh apples, oranges, and mealworms were provided daily, with unlimited drinking water. During the entire experimental period, pre-defined humane endpoints were established to minimize animal suffering. Body mass was measured every three days to monitor animal health status. No birds died or required euthanasia throughout this study. All bulbuls were released back into the wild upon completion of the experiment. Sample IDs: LT group D1–D6; RT group C1–C6; HT group G1–G6. All animal procedures were approved by the Animal Ethics and Welfare Committee of Sichuan Agricultural University (approval No. 20220085).

### 2.2. Fecal Sample Collection

At the end of the exposure experiment, each bird was individually housed in a clean cage for fecal sample collection. Fecal material was transferred with sterile forceps into pre-chilled 1.5 mL centrifuge tubes placed on ice every 10 min, with new tubes replaced every 30 min. All samples were immediately stored at −80 °C for subsequent analyses.

### 2.3. DNA Extraction

Total fecal DNA was extracted using the PowerSoil DNA Isolation Kit (MO BIO Laboratories, Carlsbad, CA, USA) according to the manufacturer’s instructions. DNA concentration was quantified using a Qubit fluorometer (Thermo Fisher Scientific, Wilmington, DE, USA). DNA purity was evaluated by the NanoDrop spectrophotometer (Thermo Fisher Scientific, Wilmington, DE, USA) based on the A260/A280 ratio, and DNA integrity was assessed via 1% agarose gel electrophoresis. Qualified DNA samples were stored at −20 °C for further use.

### 2.4. PCR Amplification and Sequencing

The V3–V4 hypervariable region of the bacterial 16S rRNA gene was amplified using universal primers 341F (5′-CCTAYGGGRBGCASCAG-3′) and 806R (5′-GGACTACNNGGGTATCTAAT-3′). PCR amplicons were purified with the Agencourt AMPure XP nucleic-acid purification kit (Beckman Coulter, Brea, CA, USA). All wet-laboratory work, including DNA detection, PCR amplification, and library construction, was performed by Novogene Bioinformatics Technology Co., Ltd., Beijing, China. Sequencing was carried out on the Illumina NovaSeq 6000 platform (PE250). Downstream bioinformatic analyses were conducted independently by our research team.

### 2.5. Data Processing and Analysis

Raw paired-end FASTQ reads were generated on the Illumina NovaSeq 6000 platform (PE250) by Novogene. Quality control, denoising, merging, and ASV inference were carried out using the DADA2 package (v1.30.0). Quality filtering was performed and PhiX contaminant sequences were removed; no mandatory read-length truncation was applied. Primers had been removed by the sequencing company, and residual primer contamination was verified to be negligible. Error-rate learning, dereplication, denoising, read merging (minOverlap = 2), and chimera removal were implemented to generate the ASV abundance table. Taxonomic assignment was performed against the SILVA v138.1 reference database. Sequences assigned to chloroplasts, mitochondria, archaea, and ASVs unclassified at the phylum level were filtered out.

To mitigate biases caused by uneven sequencing depth, all samples were rarefied to the minimum sequencing depth prior to downstream diversity analyses. Alpha-diversity metrics included observed ASVs (based on the rarefied dataset), the Shannon index, Simpson index, Pielou’s evenness (based on relative abundances), and Faith’s PD (computed from an ASV-derived neighbor-joining phylogenetic tree). For beta-diversity, principal-coordinate analysis (PCoA) was conducted based on the Aitchison distance, and non-metric multidimensional scaling (NMDS) using the Bray–Curtis distance was applied for complementary validation. Permutational multivariate analysis of variance (PERMANOVA, adonis2, 999 permutations) was employed to test differences in community composition among groups, and the permutational multivariate dispersion test (PERMDISP, betadisper) was used to examine homogeneity of group dispersions. Distances from each sample to its group centroid were calculated and compared using the Kruskal–Wallis test.

Taxonomic bar plots at phylum and genus levels were visualized on the Wekemo Bioincloud platform (https://www.bioincloud.tech/, accessed 2 November 2026). Alpha-diversity, beta-diversity, and linear discriminant-analysis effect-size (LEfSe) analyses were implemented in R. Group-wise comparisons of relative abundance at phylum and genus levels were conducted using the Kruskal–Wallis test followed by Benjamini–Hochberg-corrected Dunn’s post hoc test. LEfSe was run via the microbiomeMarker package (v1.13.2), with *α* = 0.05 for both Kruskal–Wallis and Wilcoxon tests and an LDA score threshold > 2.0. Functional prediction was carried out using PICRUSt2 on the Wekemo Bioincloud platform. Comparisons of KEGG pathways (Level 2 and Level 3) were performed with STAMP (v2.1.3) on the Tutuyun cloud platform (https://www.cloudtutu.com/, accessed 2 November 2026), employing Welch’s *t*-test with Benjamini–Hochberg FDR correction (*q* < 0.05).

All statistical analyses were performed in R (v4.5.1). The main R packages used were DADA2 (v1.30.0), vegan (v2.6-4), phyloseq (v1.46.0), picante (v1.8.2), phangorn (v2.11.1), DECIPHER (v2.28.0), microbiomeMarker (v1.13.2), and FSA (v0.9.5). Raw sequencing data have been deposited in the NCBI Sequence Read Archive (SRA) under accession PRJNA1533887.

## 3. Results

Following quality-control, denoising, read merging, chimera removal, and filtering of chloroplasts, mitochondria, archaea, and phylum-level unclassified taxa, a total of 1490 ASVs were retained for downstream analyses. To correct for uneven sequencing depth, all samples were rarefied to 4323 reads, yielding a Good’s coverage of 99.99%. Rarefaction curves approached saturation across all samples (Supplementary Figure S1), indicating that the sequencing depth captured the vast majority of bacterial diversity.

### 3.1. Effects of Temperature on Bacterial Community Composition

**Figure 1:**
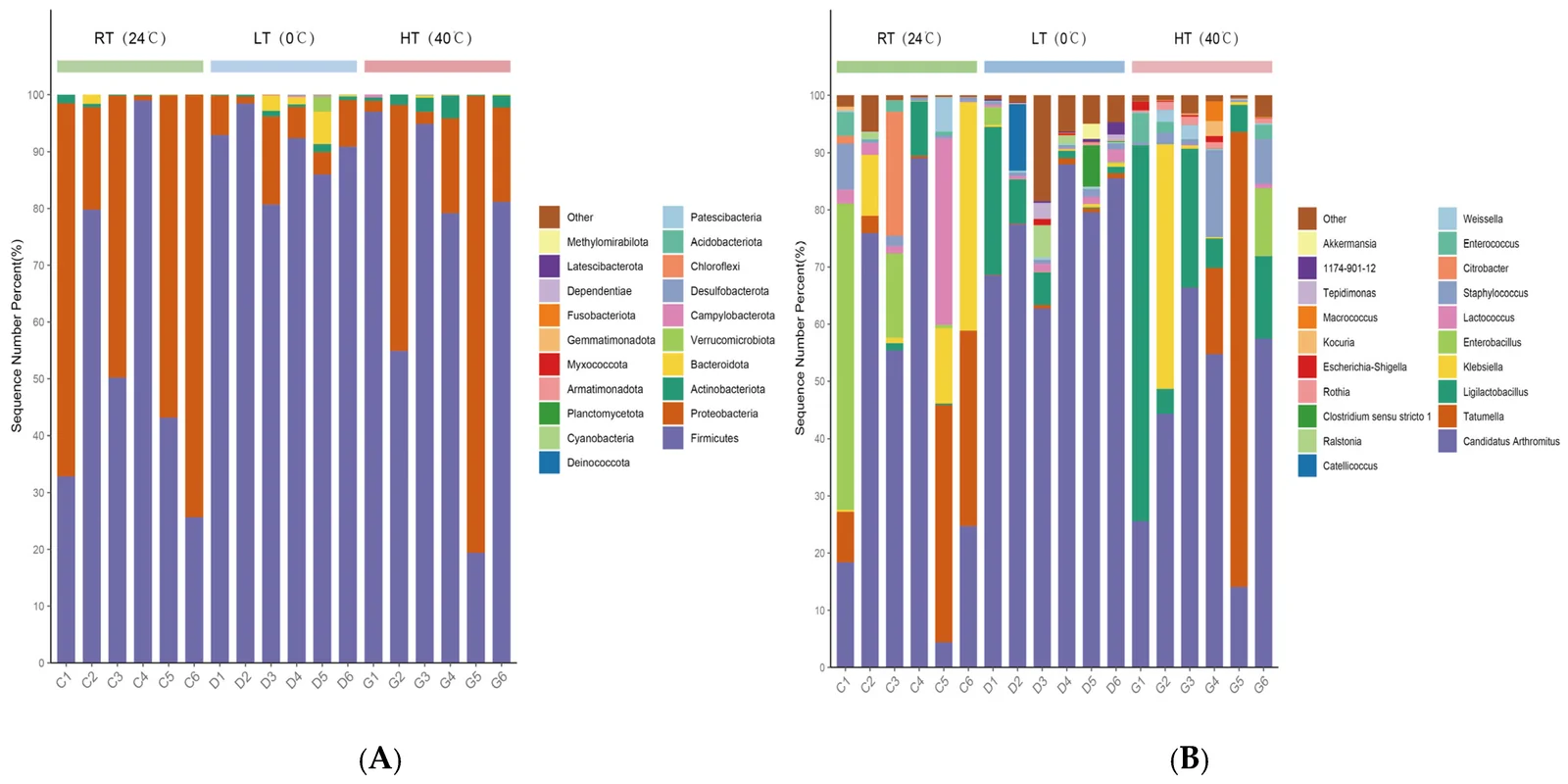
Microbial community composition under different temperature treatments. (**A**) Stacked bar chart illustrating microbial community composition at the phylum level; (**B**) stacked bar chart illustrating microbial community composition at the genus level. C1–C6 correspond to the room-temperature group (RT, 24 °C); D1–D6 correspond to the low-temperature group (LT, 0 °C); G1–G6 correspond to the high-temperature group (HT, 40 °C). The *y*-axis indicates the sequence number percentage (%). “Others” indicates taxa with relative abundance below 1%.

Taxonomic composition analysis showed that a total of 23 bacterial phyla were annotated. Firmicutes and Proteobacteria were the dominant phyla across all samples, together accounting for more than 90% of the relative abundance in each sample (Figure 1A). Firmicutes was dominant in all groups, with the highest mean relative abundance in the LT group (90.2%), followed by the HT group (71.1%) and the RT group (55.1%); however, the Kruskal–Wallis test showed that the inter-group difference was not statistically significant (*χ*^2^ = 3.825, df = 2, *p* = 0.148; Supplementary Table S1). Proteobacteria showed the opposite trend, with the highest relative abundance in the RT group (44.2%), followed by the HT group (26.8%), and the lowest in the LT group (6.9%); the inter-group difference was also not statistically significant (*χ*^2^ = 4.257, df = 2, *p* = 0.119; Supplementary Table S1).

At the genus level, a total of 327 bacterial genera were annotated. *Candidatus Arthromitus* was the most abundant genus in all groups (Figure 1B), with relative abundances of 42.54% in the RT group, 75.39% in the LT group, and 43.30% in the HT group. The Kruskal–Wallis test revealed a significant overall difference among groups in the relative abundance of *Candidatus Arthromitus* (*χ*^2^ = 6.468, df = 2, *p* = 0.039), but Dunn’s post hoc test detected no significant difference between any pair of groups (adjusted *p* > 0.05; Supplementary Table S1). The relative abundances of *Ligilactobacillus* in the RT, LT, and HT groups were 1.78%, 6.83%, and 19.68%, respectively. The Kruskal–Wallis test indicated a significant overall difference among groups in the relative abundance of *Ligilactobacillus* (*χ*^2^ = 6.884, df = 2, *p* = 0.032). Dunn’s post hoc multiple-comparison test showed that a significant difference existed only between the RT and HT groups (adjusted *p* = 0.028), whereas the differences between the RT and LT groups and between the LT and HT groups were not significant (adjusted *p* = 0.157 and 0.330, respectively; Supplementary Table S4). *Tatumella* was relatively abundant in the RT and HT groups (14.35% and 15.74%), whereas its relative abundance was only 0.56% in the LT group; the inter-group difference was not statistically significant (*χ*^2^ = 1.584, df = 2, *p* = 0.453; Supplementary Table S4). The full relative-abundance data and statistical test results at the genus level are provided in Supplementary Table S1.

### 3.2. Effects of Temperature Stress on Alpha and Beta Diversity of the Gut Microbiota

**Figure 2:**
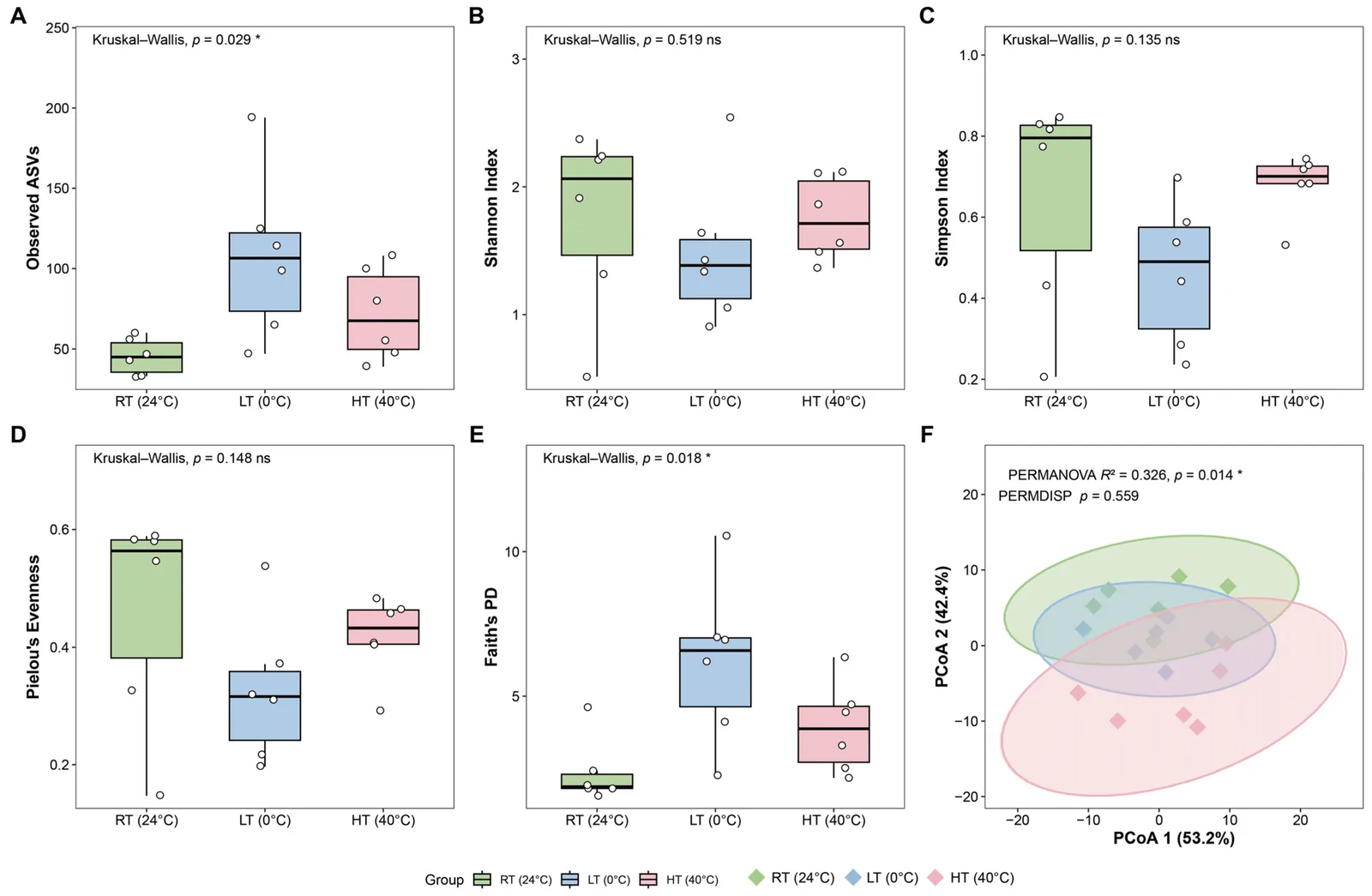
Alpha and beta diversity of the gut microbiota under three temperature treatments. (**A**) Observed ASVs. (**B**) Shannon index. (**C**) Simpson index. (**D**) Pielou’s evenness. (**E**) Faith’s phylogenetic diversity (Faith’s PD). (**F**) Principal coordinate analysis (PCoA) based on Aitchison distances at the genus level. Boxplots show the median (horizontal line), interquartile range (box), and individual data points (circles). *p* values were calculated using Kruskal–Wallis tests for panels A–E; * *p* < 0.05; ns, not significant (*p* ≥ 0.05). In panel F, the PERMANOVA *R*^2^ *p* value and the PERMDISP *p* value are annotated. Ellipses represent 95% confidence intervals. Colors indicate temperature groups: green, RT (24 °C); blue, LT (0 °C); pink, HT (40 °C). *n* = 6 birds per group. RT, room temperature; LT, low temperature; HT, high temperature.

To explore the effects of temperature treatment on the diversity of the gut microbial community in light-vented bulbuls, we performed alpha and beta diversity analyses (Figure 2A–F). For alpha diversity analysis, observed ASVs and Faith’s phylogenetic diversity (Faith’s PD) were used to evaluate community species richness, while the Shannon, Simpson, and Pielou evenness indices were calculated. As the data did not follow a normal distribution, the Kruskal–Wallis test was adopted for inter-group comparisons. For indices with significant overall differences, post hoc pairwise comparisons were further performed using Benjamini–Hochberg-corrected Dunn’s test.

The results showed that the species-richness indices observed ASVs and Faith’s PD exhibited significant inter-group differences (observed ASVs: *χ*^2^ = 7.07, df = 2, *p* = 0.029, Figure 2A; Faith’s PD: *χ*^2^ = 8.05, df = 2, *p* = 0.018, Figure 2E). The LT group had the highest median richness (observed ASVs: 106.5, IQR 73.5–122.2; Faith’s PD: 6.58, IQR 4.64–7.03), higher than the corresponding values in the RT group (observed ASVs: 45.0, IQR 35.5–53.8; Faith’s PD: 1.87, IQR 1.81–2.30) and the HT group (observed ASVs: 67.5, IQR 49.8–95.0; Faith’s PD: 3.87, IQR 2.71–4.65). No significant inter-group differences were detected for the Shannon, Simpson, and Pielou evenness indices (Shannon: *χ*^2^ = 1.31, df = 2, *p* = 0.519, Figure 2B; Simpson: *χ*^2^ = 4.01, df = 2, *p* = 0.135, Figure 2C; Pielou: *χ*^2^ = 3.83, df = 2, *p* = 0.148, Figure 2D), with only minor numerical fluctuations among the three groups. The RT group displayed a wider intra-group distribution range for the Shannon and Simpson indices, suggesting greater inter-individual variation within this group. Full Kruskal–Wallis statistics and median (IQR) values for each group are provided in Supplementary Tables S2 and S3. Taken together, temperature mainly affected the species richness and phylogenetic diversity of gut communities, whereas no significant inter-group differences were detected in community evenness.

Principal coordinate analysis (PCoA) was performed based on the Aitchison distance matrix at the genus level to resolve overall structural differences in gut microbial communities among the three temperature-treatment groups. The first two axes of PCoA, PCoA1 and PCoA2, explained 53.2% and 42.4% of the total variation, respectively (Figure 2F). Non-metric multidimensional scaling (NMDS) based on the Bray–Curtis distance further illustrated the pattern of community differentiation (stress = 0.108, Supplementary Figure S2). PERMANOVA revealed significant differences in gut microbial community structure among temperature-treatment groups (*R*^2^ = 0.326, *p* = 0.014, Figure 2F). PERMDISP based on the Aitchison distance showed no significant difference in intra-group dispersion across the three groups (*p* = 0.559, Figure 2F). We further calculated the distance from each sample to its corresponding group centroid: under the Bray–Curtis distance, the Kruskal–Wallis test indicated a significant inter-group difference (*p* = 0.013, Supplementary Figure S3), with higher intra-group dispersion in the RT and HT groups than in the LT group. This difference pattern was only obvious under the Bray–Curtis distance and was not significant under the Aitchison distance, suggesting that the intra-group dispersion difference observed with Bray–Curtis may be confounded by the compositional nature and sparsity of the data. Full results of pairwise PERMANOVA and PERMDISP are listed in Supplementary Table S4. In summary, temperature treatment significantly altered the overall composition of the gut microbial community in light-vented bulbuls. Since PERMDISP was non-significant, the observed community difference was not driven by differences in intra-group dispersion and more likely reflected shifts in community centroids.

### 3.3. Enrichment of Temperature-Responsive Differential Gut Microbial Taxa and Their Functional Traits

**Figure 3:**
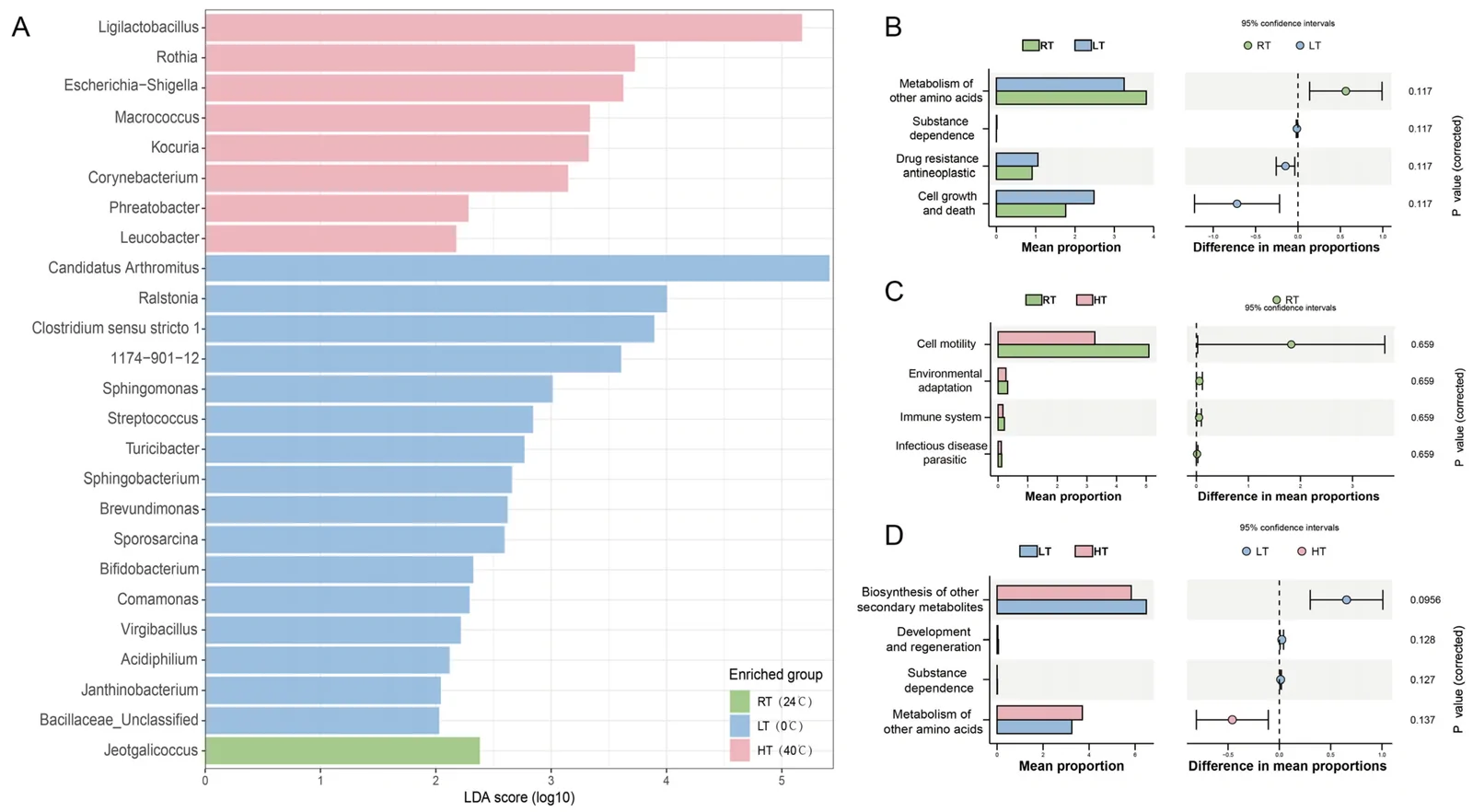
Differential taxa and predicted functional profiles among temperature treatments. (**A**) LEfSe analysis of differentially abundant genera (Kruskal–Wallis *p* < 0.05, Wilcoxon *p* < 0.05, LDA > 2.0). Bars represent LDA scores (log10); colors indicate the enriched group: green, RT (24 °C); blue, LT (0 °C); pink, HT (40 °C). (**B**–**D**) STAMP analysis of PICRUSt2-predicted KEGG pathways at Level 2 for pairwise comparisons: (**B**) RT vs. LT, (**C**) RT vs. HT, and (**D**) LT vs. HT. Extended error bar plots show mean proportion differences with 95% confidence intervals (Welch’s *t*-test, Benjamini–Hochberg FDR correction, *q* < 0.05). The dashed vertical line indicates zero difference in mean proportions between the two groups. Adjusted *p* values are shown on the right. No pathway showed a significant difference after FDR correction (all *q* > 0.05). n = 6 birds per group. RT, room temperature; LT, low temperature; HT, high temperature.

To identify bacterial genera with significantly different abundances under different temperature treatments, we performed LEfSe analysis (Kruskal–Wallis *α* = 0.05, Wilcoxon *α* = 0.05, LDA > 2.0), and the results are shown in Figure 3A. A total of 25 differentially abundant genera were identified, among which 16 were enriched in the LT group, eight in the HT group, and one in the RT group. The taxa with the highest LDA scores in the LT group were Candidatus *Arthromitus* (LDA = 5.45), *Ralstonia* (LDA = 4.00), *Clostridium* sensu stricto 1 (LDA = 3.89), and *Sphingomonas* (LDA = 3.01). The top biomarker genera in the HT group with the highest LDA scores were *Ligilactobacillus* (LDA = 5.18), *Rothia* (LDA = 3.73), and *Escherichia-Shigella* (LDA = 3.63). Only one genus, *Jeotgalicoccus* (LDA = 2.38), was enriched in the RT group. The full list of biomarker taxa and their LDA scores are provided in Supplementary Table S5.

Based on KEGG pathway abundances predicted by PICRUSt2, pairwise comparisons among the three temperature-treatment groups were conducted using STAMP (Welch’s *t*-test, Benjamini–Hochberg FDR correction, *q* < 0.05). At KEGG Level 2, no significantly differentially abundant pathways were detected in pairwise comparisons of RT vs. LT, RT vs. HT, and LT vs. HT (all *q* > 0.05; Figure 3B–D). Consistent results were obtained when repeating the analysis at KEGG Level 3; no significantly differentially abundant pathways were identified (all *q* > 0.05; Supplementary Figures S4–S6). The mean weighted NSTI value for PICRUSt2 predictions was 0.047 (range: 0.017–0.102), indicating high reliability of the functional predictions. Taken together, although temperature treatment induced abundance shifts in some microbial taxa at the genus level, no significant differences in KEGG pathways were detected using 16S rRNA-gene-based functional prediction.

## 4. Discussion

This controlled indoor experiment suggests that temperature can alter the overall community structure of gut bacteria in *Pycnonotus sinensis*, with elevated microbial richness under low-temperature conditions [17,18,25]. Community compositional changes occur mainly at the genus level, while core phylum-level microbiota remain largely unaltered. These results complement field surveys of avian microbiota along seasonal and geographical gradients [25,32,37] and help clarify temperature-related host–microbe interactions in passerines [22,23,24].

Firmicutes and Proteobacteria dominated all samples, together accounting for more than 90% of total bacterial abundance, consistent with the general composition of avian gut microbiota [22,23,24,38]. No significant differences were detected between groups for these two phyla. Together with the non-significant evenness indices, this suggests that temperature treatment did not disrupt the core phylum-level microbial framework; its filtering effect acted mainly on moderate- to low-abundance taxa. High-abundance core avian gut microbes can buffer environmental perturbations, and community responses may be more detectable at finer taxonomic levels, such as the genus and ASV [24,39,40]. Our data therefore support analyzing genus- and ASV-level responses when assessing temperature stress on the gut microbiota of *P. sinensis*; phylum-level data alone may miss part of the community response [39,40,41].

Alpha diversity results showed that observed ASVs and Faith’s PD were higher in the low-temperature group than in the room- and high-temperature groups, whereas evenness-related indices did not differ statistically among groups. This indicates that low temperatures mainly affected the detection of rare taxa without changing the relative abundance of dominant taxa [17,32]. Increased richness under winter conditions has also been reported in field surveys of passerines [32]. Because diet and light were standardized in this experiment, the results suggest that temperature was an important driver of this diversity pattern [14,25]. Higher richness may expand the functional repertoire of gut microbes and help hosts cope with physiological challenges at low temperatures [18,19]. However, higher ASV counts may also include transient environmental microbes and cannot be directly interpreted as evidence for host adaptation [42,43].

Genus-level biomarkers identified by LEfSe provide clues for interpreting microbial responses. *Candidatus Arthromitus* reached its highest relative abundance in the LT group (75.39%) and was identified by LEfSe as an LT-enriched biomarker (LDA = 5.45), although pairwise Dunn’s post hoc comparisons did not remain significant after correction; this taxon is known to contribute to intestinal immune homeostasis in vertebrates [44]. Given the enhanced thermogenic metabolism of *P. sinensis* under cold exposure [3,4,14,15,36], we speculate that its numerical increase may be associated with changes in host immunity and energy metabolism under cold stress, although this interpretation requires further verification. *Ligilactobacillus*, enriched in the high-temperature group, can ferment carbohydrates and produce short-chain fatty acids [45,46]. Some closely related strains are heat-tolerant [47], suggesting that its enrichment may reflect a microbial response to altered intestinal conditions under high temperatures. *Tatumella* was more abundant in the room- and high-temperature groups. This genus is often associated with plants and fresh fruits, and its pattern may reflect food-borne microbial input [42]. *Rothia* and *Kocuria* recovered from high-temperature samples are common environmental and skin-associated potential contaminants. Because no extraction or PCR negative controls were included, we cannot fully distinguish true gut symbionts from exogenous signals introduced during sample processing. This methodological concern is common in fecal microbiome studies of wild birds [23,43].

PERMANOVA based on Aitchison distance showed that temperature treatment significantly altered the overall gut microbial community structure. A non-significant PERMDISP result suggests that group separation was driven more by centroid displacement than by differences in within-group dispersion [40]. The room-temperature group had a wider range of alpha-diversity values and greater inter-individual microbial variation. Wild bird gut microbiota often show high inter-individual variability [22,23,24,43]. Without directional filtering by thermal stress, intrinsic baseline differences among captive birds may become more apparent [39,43]. In contrast, high-temperature, and especially low-temperature, conditions imposed stronger environmental filtering, compressing inter-individual variation and driving within-group convergence [17,18]. LEfSe identified 25 differential biomarkers, 16 of which were in the low-temperature group. This suggests that low temperatures have greater potential to remodel the gut microbiota of *P. sinensis* than high temperatures [19,48]. Several environmental or plant-associated genera, including *Ralstonia*, *Clostridium* sensu stricto 1, and *Sphingomonas*, were enriched at low temperature. This implies that the contribution of environmental and plant-derived microbes to gut communities may shift under cold conditions [32,42]. Distinguishing transient allochthonous microbes from true resident symbionts remains an important issue in wild bird microbiome research [23,24], and correlative shifts should not be overinterpreted as evidence of host adaptive evolution [49].

After characterizing taxon-level divergence, we used PICRUSt2 for functional prediction. No KEGG Level 2 or Level 3 pathways differed significantly after multiple-testing correction [34]. Although the mean NSTI was below 0.15, supporting basic prediction reliability, 16S rRNA-based functional prediction reflects community genetic potential rather than actual in vivo metabolic activity [34,40]. Functional redundancy is common in microbial communities, so taxonomic turnover does not necessarily produce detectable pathway-level changes [40]. The limited sample size per group further reduced statistical power [41]. These functional results are therefore exploratory and hypothesis-generating and require validation with metagenomics or metabolomics [34].

This study has several limitations that should be considered. First, the sample size was relatively small, and no pre-treatment baseline fecal samples were collected. The birds were wild-caught and kept in captivity; captivity itself can reshape avian gut microbiota, making it difficult to separate baseline individual differences from captive-housing effects [23,39,43]. In addition, each temperature treatment was maintained in a separate chamber, so temperature effects may be confounded by chamber-specific conditions such as humidity, airflow, and food spoilage rates [50]. Second, no negative controls were included; fecal sampling cannot fully exclude exogenous contamination, and the origin of some differential taxa cannot be determined with certainty [43]. Third, only fecal samples were analyzed, which reflect the distal gastrointestinal tract. Host physiological indices such as corticosterone, body mass change, and basal metabolic rate were not measured. We therefore cannot distinguish adaptive host plasticity from passive perturbation caused by thermal stress [3,4,14]. Consequently, the microbial changes reported here are correlative, and we cannot demonstrate that gut microbiota mediate thermal adaptation in *P. sinensis* [17,18,49]. Fourth, 16S amplicon data have inherent compositional properties: a large change in one taxon mathematically alters the relative abundances of all other taxa, complicating interpretation [40,41].

To address these limitations, future work should increase sample sizes, collect pre-treatment baseline samples, and include comprehensive negative controls to reduce confounding [41,43]. Measuring host physiological phenotypes concurrently would help separate direct temperature filtering from host-mediated indirect effects [3,14]. Multi-omics approaches could resolve actual microbial functions [34]. Seasonal field sampling would also help assess whether laboratory findings apply to wild populations [25,32]. In summary, this controlled experiment suggests that temperature can alter gut microbial richness and community structure in *P. sinensis*, with stronger remodeling under low temperature. It provides laboratory evidence for understanding host–microbe interactions in passerines under climate change [1,17,24]. Future studies integrating host physiology and multi-omics approaches will help clarify whether the microbial changes observed here contribute to physiological and thermal adaptation in *P. sinensis* and in allied species affected by climate change.

## 5. Conclusions

This study investigated the effects of long-term temperature exposure on fecal gut microbiota in light-vented bulbuls (*Pycnonotus sinensis*) using controlled indoor manipulations. Temperature treatment significantly reshaped the overall gut microbial community structure. Low-temperature exposure increased species richness and phylogenetic diversity without altering community evenness. Temperature-driven filtering mainly operated at the genus level, while core phylum-level microbiota remained stable, and LEFSe identified 16 LT-enriched genera versus eight HT-enriched genera, indicating that low temperatures were associated with a broader set of differentially abundant genera. Although numerous differentially enriched genera were identified, no statistically significant KEGG pathway differences were detected after correction based on 16S rRNA-derived functional prediction. This study provides experimental evidence for temperature-dependent host–gut microbial responses in passerines under climate change. Further integration of host physiological phenotyping and multi-omics analyses will help disentangle the mechanisms underlying temperature-mediated host–microbe interactions.

## Data Availability

The 16S rRNA gene sequencing data generated in this study have been deposited in the National Center for Biotechnology Information database under accession code PRJNA1533887 (https://www.ncbi.nlm.nih.gov/bioproject/PRJNA1533887, 8 November 2026).

## Supplementary Materials

The following supporting information can be downloaded at: https://www.mdpi.com/article/10.3390/biology15212045/s1, Figure S1: Rarefaction curves of the 18 faecal samples; Figure S2: Non-metric multidimensional scaling (NMDS) ordination based on Bray–Curtis distances; Figure S3: Distance from each sample to its group centroid based on Bray–Curtis distances; Figure S4: STAMP analysis of PICRUSt2-predicted KEGG pathways at Level 3 for RT vs. LT; Figure S5: STAMP analysis of PICRUSt2-predicted KEGG pathways at Level 3 for RT vs. HT; Figure S6: STAMP analysis of PICRUSt2-predicted KEGG pathways at Level 3 for LT vs. HT; Table S1: Kruskal–Wallis and Dunn post-hoc test results for phylum- and genus-level relative abundances; Table S2: Kruskal–Wallis statistics for alpha-diversity indices; Table S3: Median and interquartile range (IQR) of alpha-diversity indices for each group; Table S4: Global and pairwise PERMANOVA and PERMDISP results; Table S5: LEfSe biomarker taxa and their LDA scores.

## References

[1] Ingala M.R., Albert L., Addesso A., Watkins M.J., Knutie S.A. (2021). Differential effects of elevated nest temperature and parasitism on the gut microbiota of wild avian hosts. Anim. Microbiome.

[2] Dietz M.W., Matson K.D., Versteegh M.A., Van Der Velde M., Parmentier H.K., Arts J.A., Salles J.F., Tieleman B.I. (2022). Gut microbiota of homing pigeons shows summer-winter variation under constant diet indicating a substantial effect of temperature. Anim. Microbiome.

[3] Liukkonen M., Muriel J., Martínez-Padilla J., Nord A., Pakanen V.M., Rosivall B., Tilgar V., van Oers K., Grond K., Ruuskanen S. (2024). Seasonal and environmental factors contribute to the variation in the gut microbiome: A large-scale study of a small bird. J. Anim. Ecol..

[4] Bo T., Song G., Zhang M., Xu X., Duan J., She H., Fang Y., Li W., Wen J., Liu J. (2025). Gut microbiota contribute to high-altitude adaptation in tree sparrows. mSystems.

[5] Bodawatta K.H., Hird S.M., Grond K., Poulsen M., Jønsson K.A. (2022). Avian gut microbiomes taking flight. Trends Microbiol..

[6] Nearing J.T., Douglas G.M., Hayes M.G., MacDonald J., Desai D.K., Allward N., Jones C.M.A., Wright R.J., Dhanani A.S., Comeau A.M. (2022). Microbiome differential abundance methods produce different results across 38 datasets. Nat. Commun..

[7] Lyte J.M., Jia X., Caputi V., Zhang D., Daniels K.M., Phillips G.J., Lyte M. (2025). Heat stress in chickens induces temporal changes in the cecal microbiome concomitant with host enteric serotonin responses. Poult. Sci..

[8] Sepulveda J., Moeller A.H. (2020). The Effects of Temperature on Animal Gut Microbiomes. Front. Microbiol..

[9] Khakisahneh S., Zhang X.Y., Nouri Z., Wang D.H. (2020). Gut Microbiota and Host Thermoregulation in Response to Ambient Temperature Fluctuations. mSystems.

[10] Carey H.V., Walters W.A., Knight R. (2013). Seasonal restructuring of the ground squirrel gut microbiota over the annual hibernation cycle. Am. J. Physiol. Regul. Integr. Comp. Physiol..

[11] Grond K., Sandercock B.K., Jumpponen A., Zeglin L.H. (2018). The avian gut microbiota: Community, physiology and function in wild birds. J. Avian Biol..

[12] Wu Y., Xiong Y., Ji Y., Cheng Y., Zhu Q., Jiao X., Chang Y., Zhao N., Yang J., Lei F. (2024). Metabolic and microbial changes in light-vented bulbul during recent northward range expansion. Curr. Zool..

[13] Zheng W.H., Liu J.S., Swanson D.L. (2014). Seasonal phenotypic flexibility of body mass, organ masses, and tissue oxidative capacity and their relationship to resting metabolic rate in Chinese bulbuls. Physiol. Biochem. Zool..

[14] Douglas G.M., Maffei V.J., Zaneveld J.R., Yurgel S.N., Brown J.R., Taylor C.M., Huttenhower C., Langille M.G.I. (2020). PICRUSt2 for prediction of metagenome functions. Nat. Biotechnol..

[15] Hedblom G.A., Reiland H.A., Sylte M.J., Johnson T.J., Baumler D.J. (2018). Segmented Filamentous Bacteria-Metabolism Meets Immunity. Front. Microbiol..

[16] Maniee S.A., Mahmoodian S., Zamani Amirzakaria J., Meimandipour A., Shariati V., Tavakol E. (2025). Whole-genome analysis of *Ligilactobacillus salivarius* L33, a potential probiotic strain isolated from chicken gastrointestinal tract. Microbiol. Spectr..

[17] Huo Y., Gao W., Sun X., Li Z., Dai M., Zhang S. (2025). Multiple stress pretreatments enhance the heat resistance of Ligilactobacillus salivarius SS1. Food Biosci..

[18] Wang X.J., Feng J.H., Zhang M.H., Li X.M., Ma D.D., Chang S.S. (2018). Effects of high ambient temperature on the community structure and composition of ileal microbiome of broilers. Poult. Sci..

[19] Shi D., Bai L., Qu Q., Zhou S., Yang M., Guo S., Li Q., Liu C. (2019). Impact of gut microbiota structure in heat-stressed broilers. Poult. Sci..

[20] Bodawatta K.H., Klečková I., Klečka J., Pužejová K., Koane B., Poulsen M., Jønsson K.A., Sam K. (2022). Specific gut bacterial responses to natural diets of tropical birds. Sci. Rep..

[21] Rausch P., Basic M., Batra A., Bischoff S.C., Blaut M., Clavel T., Gläsner J., Gopalakrishnan S., Grassl G.A., Günther C. (2016). Analysis of factors contributing to variation in the C57BL/6J fecal microbiota across German animal facilities. Int. J. Med. Microbiol..

[22] McKechnie A.E. (2008). Phenotypic flexibility in basal metabolic rate and the changing view of avian physiological diversity: A review. J. Comp. Physiol. B.

[23] Whitfield M.C., Smit B., McKechnie A.E., Wolf B.O. (2015). Avian thermoregulation in the heat: Scaling of heat tolerance and evaporative cooling capacity in three southern African arid-zone passerines. J. Exp. Biol..

[24] Swanson D.L., Olmstead K.L. (1999). Evidence for a Proximate Influence of Winter Temperature on Metabolism in Passerine Birds. Physiol. Biochem. Zool..

[25] Lin L., Cao M., Hu Y., Huang L., Li Z., Liu J. (2014). Effect of environmental temperature on thermogenesis and evaporative water loss in Chinese bulbuls (*Pycnonotus sinensis*). Acta Ecol. Sin..

[26] McKechnie A.E., Wolf B.O. (2019). The Physiology of Heat Tolerance in Small Endotherms. Physiology.

[27] Gerson A.R., McKechnie A.E., Smit B., Whitfield M.C., Smith E.K., Talbot W.A., McWhorter T.J., Wolf B.O. (2019). The functional significance of facultative hyperthermia varies with body size and phylogeny in birds. Funct. Ecol..

[28] Thomas C.D., Lennon J.J. (1999). Birds extend their ranges northwards. Nature.

[29] Chen I.-C., Hill J.K., Ohlemüller R., Roy D.B., Thomas C.D. (2011). Rapid Range Shifts of Species Associated with High Levels of Climate Warming. Science.

[30] Chen S., Chen Z., Lin H., Duan H., Yu J., He J. (2025). Chinese provincial-level new records for 96 resident bird species reveal poleward range shifts. Avian Res..

[31] Dunn O.P., Møller A.P. (2014). Changes in breeding phenology and population size of birds. J. Anim. Ecol..

[32] Crick H.Q.P., Sparks T.H. (1999). Climate change related to egg-laying trends. Nature.

[33] Wiley E.M., Ridley A.R. (2016). The effects of temperature on offspring provisioning in a cooperative breeder. Anim. Behav..

[34] Walther G.-R., Post E., Convey P., Menzel A., Parmesan C., Beebee T.J.C., Fromentin J.-M., Hoegh-Guldberg O., Bairlein F. (2002). Ecological responses to recent climate change. Nature.

[35] Hughes L. (2000). Biological consequences of global warming: Is the signal already apparent?. Trends Ecol. Evol..

[36] Dainese M., Crepaz H., Bottarin R., Fontana V., Guariento E., Hilpold A., Obojes N., Paniccia C., Scotti A., Seeber J. (2024). Global change experiments in mountain ecosystems: A systematic review. Ecol. Monogr..

[37] Carignan V., Villard M.-A. (2002). Selecting Indicator Species to Monitor Ecological Integrity: A Review. Environ. Monit. Assess..

[38] Moeller A.H., Sanders J.G. (2020). Roles of the gut microbiota in the adaptive evolution of mammalian species. Philos. Trans. R. Soc. Lond. Ser. B Biol. Sci..

[39] Ley R.E., Lozupone C.A., Hamady M., Knight R., Gordon J.I. (2008). Worlds within worlds: Evolution of the vertebrate gut microbiota. Nat. Rev. Microbiol..

[40] Cho I., Blaser M.J. (2012). The human microbiome: At the interface of health and disease. Nat. Rev. Genet..

[41] Grice E.A., Segre J.A. (2012). The Human Microbiome: Our Second Genome. Annu. Rev. Genom. Hum. Genet..

[42] Ríos-Covián D., Ruas-Madiedo P., Margolles A., Gueimonde M., De Los Reyes-Gavilán C.G., Salazar N. (2016). Intestinal Short Chain Fatty Acids and their Link with Diet and Human Health. Front. Microbiol..

[43] Chevalier C., Stojanović O., Colin D.J., Suarez-Zamorano N., Tarallo V., Veyrat-Durebex C., Rigo D., Fabbiano S., Stevanović A., Hagemann S. (2015). Gut Microbiota Orchestrates Energy Homeostasis during Cold. Cell.

[44] Tufail A., Bo T., Zhao N., Munro S.W., Khan B.N., Duan J., Jin J., Qu Y., Lei F., Song G. (2025). Exploring microbiome shifts across taxonomic and ecological groups of birds at a key stopover site in Punjab, Pakistan. Curr. Res. Microb. Sci..

[45] Waite D.W., Taylor M.W. (2015). Exploring the avian gut microbiota: Current trends and future directions. Front. Microbiol..

[46] Campos P.M., Greene E.S., Weglarz M.P., Dridi S. (2025). Effects of heat stress and depressed feed intake on the cecal and ileal microbiota of broiler chickens. Poult. Sci..

[47] Hu S.-N., Zhu Y.-Y., Lin L., Zheng W.-H., Liu J.-S. (2017). Temperature and photoperiod as environmental cues affect body mass and thermoregulation in Chinese bulbuls, *Pycnonotus sinensis*. J. Exp. Biol..

[48] Li S., Li X., Wang K., Li Y., Nagaoka K., Li C. (2023). Gut microbiota intervention attenuates thermogenesis in broilers exposed to high temperature through modulation of the hypothalamic 5-HT pathway. J. Anim. Sci. Biotechnol..

[49] Bodawatta K.H., Freiberga I., Puzejova K., Sam K., Poulsen M., Jønsson K.A. (2021). Flexibility and resilience of great tit (*Parus major*) gut microbiomes to changing diets. Anim. Microbiome.

[50] Louca S., Polz M.F., Mazel F., Albright M.B., Huber J.A., O’Connor M.I., Ackermann M., Hahn A.S., Srivastava D.S., Crowe S.A. (2018). Function and functional redundancy in microbial systems. Nat. Ecol. Evol..

