# Supplementary material for: Effects of Extreme Temperature Stress on the Gut Microbiota of Light-Vented Bulbul (Pycnonotus sinensis)
Source: Biology (Basel). 2026 Nov 9;15(21):2045. doi: 10.3390/biology15212045 (PMC13761995; doi:10.3390/biology15212045)
Supplement: Supplementary file 1 [file biology-15-02045-s001.zip › biology-4604519_Supplementary Fig S1-S6.pdf]

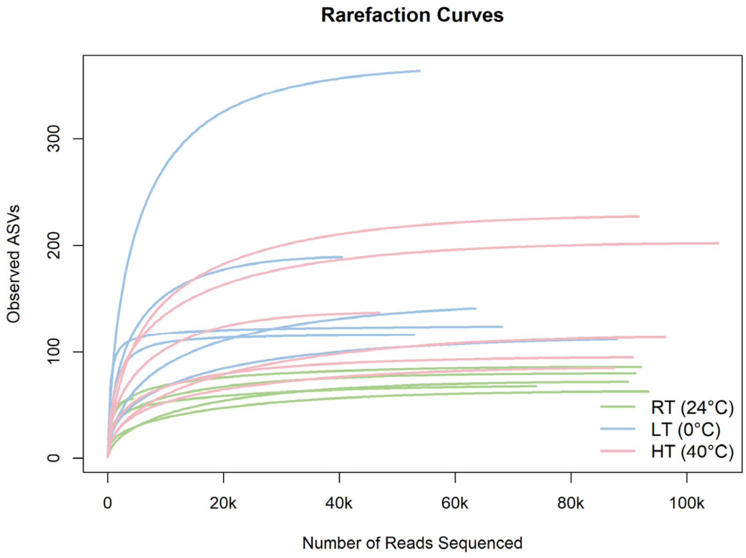

Supplementary Figure S1. Rarefaction curves of the 18 faecal samples. Each curve represents one sample, coloured by temperature group: green, RT (24 °C); blue, LT (0 °C); pink, HT (40 °C). The curves approached saturation in all samples, indicating that the sequencing depth was sufficient to capture the vast majority of bacterial diversity.

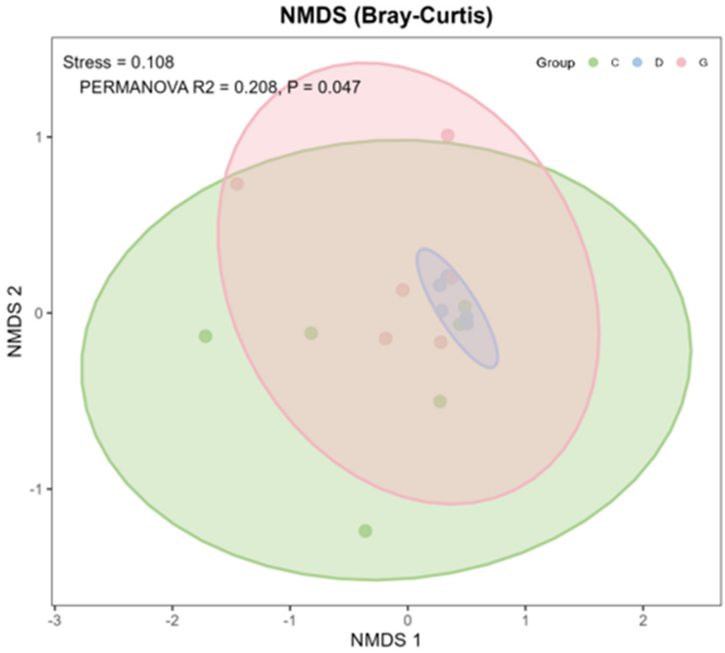

Supplementary Figure S2. Non-metric multidimensional scaling (NMDS) ordination based on Bray–Curtis distances at the genus level. Ellipses represent 95% confidence intervals. Stress value and PERMANOVA R^2 and P value are annotated. Colours indicate temperature groups: green, RT (24 °C); blue, LT (0 °C); pink, HT (40 °C).

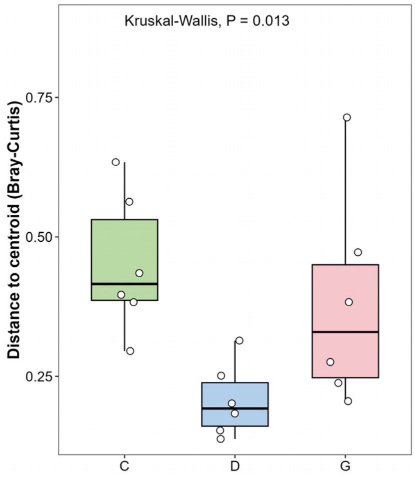

Supplementary Figure S3. Distance from each sample to its group centroid based on Bray–Curtis distances. Boxplots show the median (horizontal line), interquartile range (box), and individual data points (circles). P value was calculated using the Kruskal–Wallis test. Colours indicate temperature groups: green, RT (24 °C); blue, LT (0 °C); pink, HT (40°C).

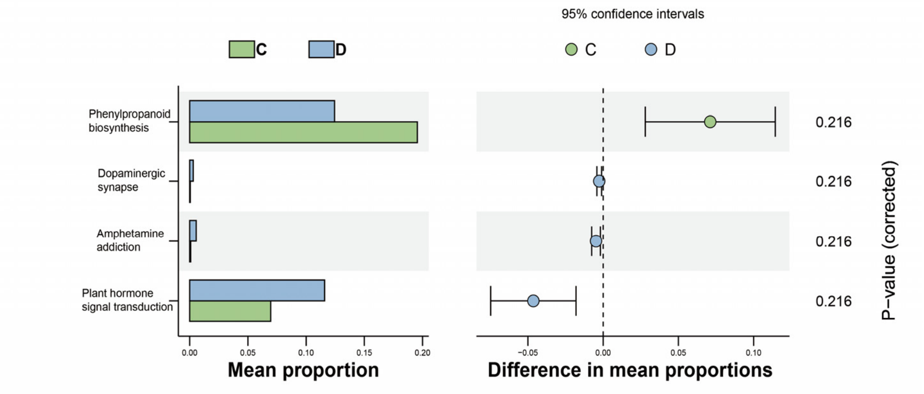

Supplementary Figure S4. STAMP analysis of PICRUSt2-predicted KEGG pathways at Level 3 for the pairwise comparison RT vs LT. Extended error bar plots show mean proportion differences with 95% confidence intervals (Welch's t-test, Benjamini–Hochberg FDR correction, $q < 0.05$). Adjusted P values are shown on the right. No pathway showed a significant difference after FDR correction (all $q > 0.05$). C, RT (24 °C); D, LT (0 °C).

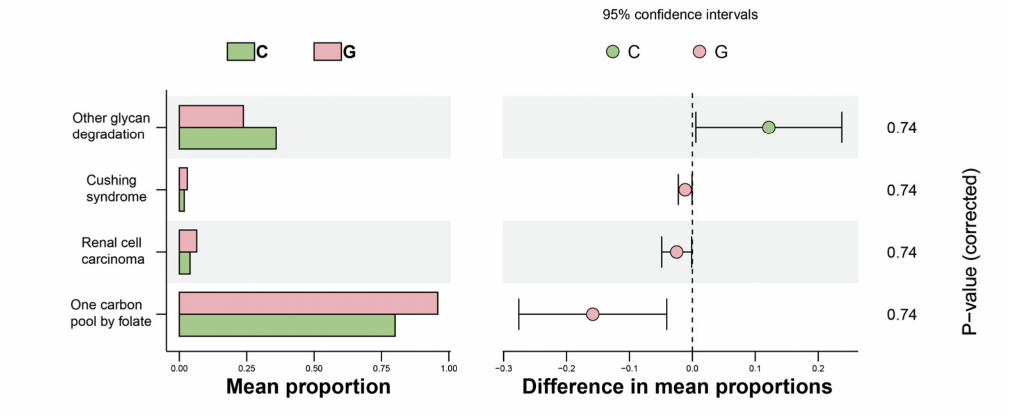

Supplementary Figure S5. STAMP analysis of PICRUSt2-predicted KEGG pathways at Level 3

for the pairwise comparison RT vs HT. Extended error bar plots show mean proportion differ ences with 95% confidence intervals (Welch's t-test, Benjamini–Hochberg FDR correction, q < 0.05). Adjusted P values are shown on the right. No pathway showed a significant difference a fter FDR correction (all q > 0.05). C, RT (24 °C); G, HT (40 °C).

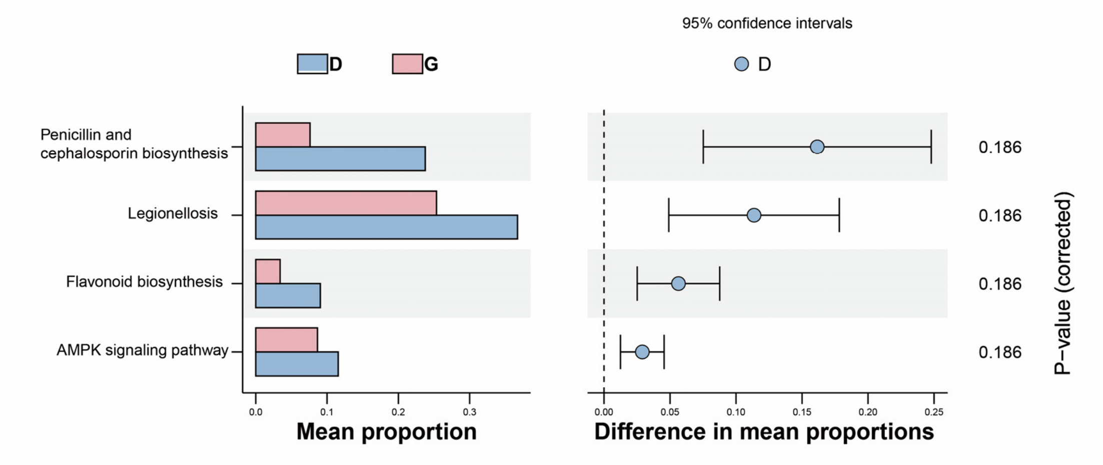

Supplementary Figure S6. STAMP analysis of PICRUSt2-predicted KEGG pathways at Level 3 for the pairwise comparison LT vs HT. Extended error bar plots show mean proportion differ ences with 95% confidence intervals (Welch's t-test, Benjamini–Hochberg FDR correction, q < 0.05). Adjusted P values are shown on the right. No pathway showed a significant difference a fter FDR correction (all q > 0.05). D, LT (0 °C); G, HT (40 °C).
